# Distinct microbial communities associated with health‐relevant wild berries

**DOI:** 10.1111/1758-2229.70048

**Published:** 2024-11-14

**Authors:** Iglė Vepštaitė‐Monstavičė, Juliana Lukša, Živilė Strazdaitė‐Žielienė, Saulius Serva, Elena Servienė

**Affiliations:** ^1^ Laboratory of Nucleic Acid Biochemistry, Institute of Biosciences, Life Sciences Center Vilnius University Vilnius Lithuania; ^2^ Laboratory of Genetics Nature Research Centre Vilnius Lithuania; ^3^ Department of Chemistry and Bioengineering, Faculty of Fundamental Sciences Vilnius Gediminas Technical University (VILNIUSTECH) Vilnius Lithuania

## Abstract

Lingonberries (*Vaccinium vitis‐idaea L*.), rowanberries (*Sorbus aucuparia L*.) and rosehips (*Rosa canina L*.) positively affect human health due to their healing properties, determined by a high content of bioactive compounds. The consumption of unprocessed wild berries is relevant and encouraged, making their in‐depth microbiological characterization essential for food safety. This study presents the first high‐throughput sequencing analysis of bacterial and fungal communities distributed on the surface of lingonberries, rowanberries and rosehips. Significant plant‐defined differences in the taxonomic composition of prokaryotic and eukaryotic microbiota were observed. The bacterial community on rosehips was shown to be prevalent by *Enterobacteriaceae*, lingonberries by *Methylobacteriaceae* and rowanberries by *Sphingomonadaceae* representatives. Among the fungal microbiota, Dothioraceae dominated on rosehips and Exobasidiaceae on lingonberries; meanwhile, rowanberries were inhabited by a similar level of a broad spectrum of fungal families. Cultivable yeast profiling revealed that lingonberries were distinguished by the lowest amount and most distinct yeast populations. Potentially pathogenic to humans or plants, as well as beneficial and relevant biocontrol microorganisms, were identified on tested berries. The combination of metagenomics and a cultivation‐based approach highlighted the wild berries‐associated microbial communities and contributed to uncovering their potential in plant health, food and human safety.

## Graphical abstract

This research presents the first high‐throughput analysis of bacterial and fungal communities on lingonberries, rowanberries and rosehips, highlighting significant plant‐specific differences. The study reveals microbial communities with potential roles in plant health, food safety and human health, identifying both beneficial and pathogenic microorganisms. These findings highlight the importance of comprehensive microbial characterization to ensure the safe consumption of unprocessed wild berries.

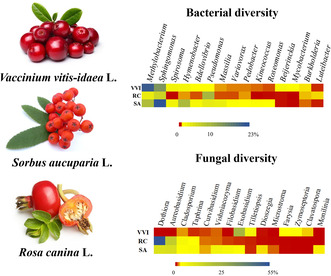

## INTRODUCTION

Recently, interest in foods and specific food components with potential health benefits has surged. Berries from diverse botanical species represent a rich reservoir of bioactive compounds with profound implications for human health and nutrition. The production of natural products delivered from berries intensified as a promising new avenue for developing antimicrobial agents and prebiotics (Samtiya et al., 2021). This interest stems from its ability to selectively hinder enteric pathogens while fostering the growth of beneficial microorganisms (Lacombe & Wu, 2017; Puupponen‐Pimiä et al., 2005). Among the wide array of wild berries, lingonberries (*Vaccinium vitis‐idaea* L.), rowanberries (*Sorbus aucuparia* L.) and rosehips (*Rosa canina* L.) are commonly found in Northern European forests and thrive in a moderately cold climate zone. These berries have gained recognition for their high content of bioactive compounds, including vitamins, particularly ascorbic acid, and have potential therapeutic properties (Cioch et al., 2017; Marungruang et al., 2020; Vilkickyte et al., 2022).

Lingonberries (also known as cowberries) are a low‐shrub plant from the *Ericaceae* family, usually harvested in the wild. Although certain cultivars are produced on a modest scale, the breeding of lingonberry plants is still in its early stages (Karlsons et al., 2021; Kowalska, 2021). These berries have exhibited remarkable beneficial properties in preventing diet‐induced obesity, enhancing insulin sensitivity and mitigating inflammation in animal models and healthy human subjects. Moreover, lingonberries have a rich history of traditional usage as a remedy for urinary tract infections, fever and rheumatism (Kowalska, 2021; Shepilov et al., 2022). Recent studies have shown that lingonberries exhibit potent antimicrobial effects, inhibiting bacteria, fungi and viral proliferation and reducing biofilm formation (Kowalska, 2021). Lingonberries extracts have antimicrobial properties against both Gram‐negative and Gram‐positive bacteria such as *Streptococcus* spp., *Porphyromonas* spp., *Salmonella* spp., *Escherichia coli*, *Serratia marcescens*, *Proteus myxofaciens*, *Micrococcus luteus*, *Bacillus subtilis*, *Clostridium* spp. and yeast *Candida* spp., *Saccharomyces cerevisiae* (Cioch et al., 2017; Pärnänen et al., 2021).

Similarly, rowanberries, alternatively known as mountain ash berries, belong to the *Rosaceae* family and typically manifest as deciduous trees reaching heights of 8–10 m (Shikov et al., 2014). Rowanberries are traditionally used for their diuretic, antioxidant, anti‐inflammatory, antidiarrheal, vasoprotective, antidiabetic and anticytotoxic properties (Bobinaitė et al., 2020; Raudonis et al., 2014). The antimicrobial activity of the extracts was observed against a broad spectrum of potentially pathogenic bacteria from *Enterococcus* spp., *Listeria* spp., *Pseudomonas* spp., *Staphylococcus* spp., and so forth genera (Aurori et al., 2024; Liepiņa et al., 2013).

The wild fruits of rosehips (*R. canina* L.), also belonging to the *Rosaceae* family, are rich in bioactive compounds like phenolic acids, proanthocyanidins and polyphenols. *R. canina* exhibits anti‐inflammatory, antioxidant, hypoglycemic and hypolipidemic properties (Golsorkhi et al., 2022). Rosehips, renowned for their exceptional vitamin C content, especially in peels, boast the highest levels of this nutrient among fruits and vegetables, ranging from 30 to 1300 mg/100 g (Ercisli, 2007). Dried rosehips are used to make tea for the treatment of cold and influenza (Winther et al., 2016). Clinical investigations have indicated that rosehip fruit powder, sold as a dietary supplement in numerous European nations, can improve osteoarthritis symptoms (Warholm et al., 2003). *R. canina* extracts exhibit activity against Gram‐positive and Gram‐negative bacteria, including *Staphylococcus aureus*, *Escherichia coli* and *Pseudomonas aeruginosa*. They have also exhibited effectiveness against yeast *Candida albicans* (Quave et al., 2008; Rovná et al., 2020).

Despite the well‐documented health benefits of lingonberries, rowanberries and rosehips, there remains a lack of knowledge about the microbial communities that colonize these berries. Both epiphytic and endophytic microorganisms play a crucial role in shaping fruits' nutritional and therapeutic properties. These microbial populations can influence plant health, resistance to pathogens, and the overall composition of bioactive compounds. Even though the previous studies have explored the antimicrobial properties of the berries themselves, only a few studies have investigated their inhabiting microbial communities, primarily using culture‐dependent methods (Maksimova et al., 2009; Nguyen et al., 2021; Rovná et al., 2015, 2020). Some culturable fungal epiphytes from *Aureobasidium*, *Cryptococcus*, *Leucosporidium*, *Metschnikowia*, *Rhodotorula* and *Sporobolomyces* genera were revealed on rowanberries (Maksimova et al., 2009). In crushed lingonberry samples, culturable fungal microorganisms representing *Aureobasidium*, *Cladosporium*, *Collophora*, *Dothidea*, *Penicillium*, *Ramularia*, *Sydowia*, *Taphrina*, *Vishniacozyma* genera were detected, along with bacteria from *Erwinia* and *Robsia* genera (Nguyen et al., 2021). From homogenized rosehips *Alternaria*, *Aspergillus*, *Candida*, *Cladosporium*, *Epicoccum*, *Phoma*, *Penicillium*, *Rhodotorula* and *Trichoderma* fungi were identified. Among bacterial genera *Clostridium*, *Aromatoleum* and *Pseudomonas* were established (Rovná et al., 2020). However, these findings are limited in scope and largely rely on culture‐based methods, which may not capture the full diversity of microbial populations on these berries. To the best of our knowledge, no reports on NGS‐based analysis of bacterial and fungal microbiota inhabiting *Vaccinium vitis‐idaea* L., *Sorbus aucuparia* L. and *Rosa canina* L. berries have been presented so far.

Considering the specific antimicrobial and health‐promoting properties of lingonberries, rowanberries and rosehips, and taking into account that cultivable microorganism profiles differ on these berries, we hypothesized that each wild berry type supports a unique microbial ecosystem. Given that the consumption of wild berries is constantly increasing, and berries‐associated microbes may observe both beneficial and adverse features, we proposed the hypothesis that wild berries‐associated microorganisms have ecological and food safety relevance. Therefore, this work aims to investigate the structure of microbial assemblages inhabiting lingonberries, rowanberries and rosehips and provide valuable insights into the importance of detected microorganisms for plant and human health. We apply metagenomics and culture‐dependent approaches to uncover the structure of microbiota and identify potential beneficial and harmful microorganisms that may influence the health‐promoting properties of tested berries. The specific objectives were: (i) to assess in‐depth bacterial assemblages on lingonberries, rosehips and rowanberries by using an NGS‐based analysis; (ii) to determine the diversity of fungal community on tested wild berries by applying metagenomics and characterize the distribution of culturable yeast and (iii) to perform a comparative analysis of microbial populations inhabiting tested wild berries and to deepen our understanding on the relevance of particular microorganisms in plant and human health. The comprehensive analysis of wild berries‐associated microorganisms provides particular insight into the structure of microbial assemblages occurring on plants in the natural environment, confers relevant information to the management of microorganisms spreading from the natural environment to agricultural ecosystems, and control of plant disease outbreaks, uncovers the potential role of microbiota in berries‐based food production and safety.

## EXPERIMENTAL PROCEDURES

### Sampling of microorganisms from the surface of berries

Lingonberries and rosehips were harvested in late August 2022, while rowanberries were sampled in mid‐September 2022 in the Vilnius district of Lithuania (Figure S1). The berries were randomly selected from three bushes and combined into one biological replicate at each location. Visually healthy fruits were aseptically collected into sterile plastic bags and processed within 2–4 h after harvesting. 300 g of fruits were placed in 500 mL of sterile 0.05 M phosphate buffer pH 6.8 and incubated at 20°C for 1 h with shaking at 120 rpm. Outwashes were filtered through 420 μm filters and centrifuged at 12,000 g for 20 min. The obtained pellet was stored at −20°C and used for microbial DNA extraction.

### DNA extraction

DNA isolation was performed from 40 mg of pellet per sample using the Genomic DNA purification kit (Thermo Fisher Scientific Baltics, Vilnius, Lithuania) and following the manufacturer's instructions. The quality and quantity parameters of the extracted DNA were measured by optical reading at 260, 280 and 234 nm, using a FastGene NanoView Photometer (Nippon Genetics Europe GmbH). The extraction efficiency and quality of total DNA were found to be specifically important for following NGS analysis.

### Bacterial and fungal DNA amplification and amplicon library preparation

DNA samples from rowanberries, rosehips and lingonberries were amplified using specific primers for fungi and bacteria. For the identification of fungal microorganisms, the ITS2 region of ribosomal DNA was amplified using primers ITS3‐KYO2 (5′‐GATGAAGAACGYAGYRAA‐3′) and ITS4 (5′‐TCCTCCGCTTATTGATATGC‐3′) (Toju et al., 2012). The V3‐V4 region of the 16S rRNA gene was amplified using a pair of 341F/785R primers (5′‐CCTACGGGNGGCWGCAG‐3′/5′‐GACTACHVGGGTATCTAATCC‐3′) (Klindworth et al., 2013) for bacteria identification. These marker genes are the most commonly used for bacteria and fungi identification, however, they are not universal and have limitations (e.g., highly conserved ITS limits the ability to distinguish closely related species or due to intraspecific variation can lead to splitting the same species) (Boers et al., 2019). Targeted amplicon libraries were prepared using Illumina adapters (www.illumina.com), validated on an Agilent Technologies Bioanalyzer DNA 1000, and sequenced in pair‐end mode on an Illumina MiSeq platform (Baseclear, Leiden, Netherlands). All sequences obtained during this work are available at the Sequence Read Archive (SRA) of the National Center for Biotechnology Information (NCBI), under accession PRJNA1128889.

### Processing and analysis of the sequencing data

The sequence data in FASTQ format were processed into the QIIME2 v2020.6 edition's built‐in commands and plugins of the QIIME2 (Bolyen et al., 2019). Briefly, amplicon primers were removed with the Cutadapt v2.8 (Martin, 2011). The paired‐end reads were denoised, merged and chimeric sequences filtered out using the DADA2 plugin (Callahan et al., 2016). For 16S multiple sequence alignment was created using MAFFT, and FastTree builds in QIIME2, both with default values. The phylogenetic tree was midpoint‐rooted. Amplicon sequencing variants (ASVs) were taxonomically classified using the Greengenes v13_5 database for bacteria and UNITE v8.3 for fungal microorganisms. The success of taxonomic representation of microorganisms heavily depends on the completeness of reference databases. Alpha diversity, representing measurements within individual samples, was calculated using rarefied ASV tables (6000 for 16S and 13,000 sequences per sample for ITS sequences per sample) (Table S1) including the Shannon index (accounting for species richness and evenness), Faith's phylogenetic diversity (which incorporates evolutionary relationships) and Pielou's evenness (a measure of how evenly species are distributed). Statistical differences in alpha diversity were tested using the non‐parametric Kruskal–Wallis test. Beta diversity, which examines the differences in microbial community composition between samples, was assessed using UniFrac distances (weighted and unweighted) for 16S data and Bray Curtis dissimilarity for ITS data. Weighted UniFrac considers the abundance of each species, while unweighted UniFrac considers the presence or absence of species, without considering abundance. Bray–Curtis dissimilarity is applied on fungal communities; this index calculates differences based on the abundance of species across samples. To test the significance of these differences, we applied PERMANOVA (Permutational Multivariate ANOVA) a non‐parametric test that assesses community structure based on 999 permutations using QIIME2 software package. The pseudo‐F statistic in PERMANOVA reflects the ratio of the variance between the groups to the variance within groups. A higher pseudo‐F value suggests a greater difference between the groups. If the pseudo‐F value is large and the *p*‐value is low, this provides strong evidence that the microbial communities in different groups are truly distinct. Beta diversity patterns were visualized using principal coordinate analysis (PCoA) based on weighted and unweighted UniFrac distance matrices.

### Cultivable yeast isolation

Approximately 40 g of rowanberries, rosehips or lingonberries were placed in 70 mL of liquid MD medium (2% dextrose, 1% (NH_4_)_2_SO_4_, 0.09% KH_2_PO_4_, 0.05% MgSO_4_, 0.023% K_2_HPO_4_, 0.01% NaCl, 0.01% CaCl_2_) and incubated for 1 h at 22°C temperature with shaking at 100 rpm. For the detection of cultivable yeasts, outwashes of berries were serially diluted in MD medium, and 100 μL aliquots were plated on YPD agar (1% yeast extract, 1% peptone, 2% dextrose, 2% agar) plates with 50 μg/mL of chloramphenicol. Experiments were performed in triplicate. Colony‐forming units per gram of berries (CFU/g) were counted. Morphologically different yeast‐like colonies were purified and identified by molecular methods. To compare the mean of CFU/g values across the three types of berries, a One‐Way Analysis of Variance (ANOVA) was conducted. Since culturable yeast recovery is prone to various biases, arising from limitations in media formulation, incubation conditions, competition with other microorganisms, and so forth therefore, the overall representation of yeast diversity is limited (Liu et al., 2022).

### Molecular identification of yeast

For the identification of cultivable yeasts, genomic DNA was isolated from freshly grown purified yeast cells using a Genomic DNA purification kit (Thermo Fisher Scientific Baltics, Vilnius, Lithuania) according to the manufacturer's instructions. PCR amplification of the region between 18S rRNA and 28S rRNA genes was implemented using ITS1 (5′‐TCCGTAGGTGAACCTGCGG‐3′) and ITS4 (5′‐TCCTCCGCTTATTGATATGC‐3′) primers, according to the following conditions: 94°C for 5 min, followed by 25 cycles of 94°C for 1 min, 53°C for 1 min 30 s and 72°C for 2 min. The final extension was performed at 72°C for 10 min. The PCR reaction mixture was performed in a total reaction of 50 μL, consisting of 5 μL DreamTaq green buffer, 1 μL of 2 mM dNTP mix, 1 μL of each primer (10 μmol/L), 2.5 units of Dream Taq DNA polymerase (all from Thermo Fisher Scientific Baltics, Vilnius, Lithuania), 1 μL of DNA template (5 ng) and sterile distilled water up to 50 μL. Amplified PCR products were purified using the GeneJet PCR purification kit (Thermo Fisher Scientific Baltics, Vilnius, Lithuania) and sequenced at BaseClear (Leiden, Netherlands). Sequencing results were compared with those offered by the FASTA network service of the EMBL‐EBI database (https://www.ebi.ac.uk/jdispatcher/sss/fasta/nucleotide).

## RESULTS

### Sequencing statistics

The bacterial and fungal communities present on lingonberries (VVI), rosehips (RC) and rowanberries (SA) were revealed by Next Generation Sequencing (NGS) of the PCR‐amplified V3‐V4 region of the 16S rRNA gene and the ITS2 region of rDNA. The total DNA was extracted from outwashes of freshly picked ripe berries. Illumina Miseq sequencing generated 2.12 million bacterial and 1.57 million fungal raw reads across 15 samples. After filtering out chimeric, mitochondrial and chloroplast sequences, the dataset revealed significant differences in amplicon sequence variants (ASVs) among the berry type. Rowanberries (SA) and lingonberries (VVI) exhibited a high number of bacterial ASVs, with 2306 and 2740 ASVs respectively. In contrast, rosehip berry (RC) samples displayed a significantly lower average ASV count of 1011. Similarly, fungal ASV counts across berry types were notably higher on rowanberry and lingonberry than on rosehip fruits. Specifically, fungal ASV counts were as follows: on rowanberries—1533, lingonberries—1502 and rosehip berries—982 (Table S1).

### The alpha diversity and beta diversity of the bacterial and fungal microbiota on berries

**FIGURE 1:**
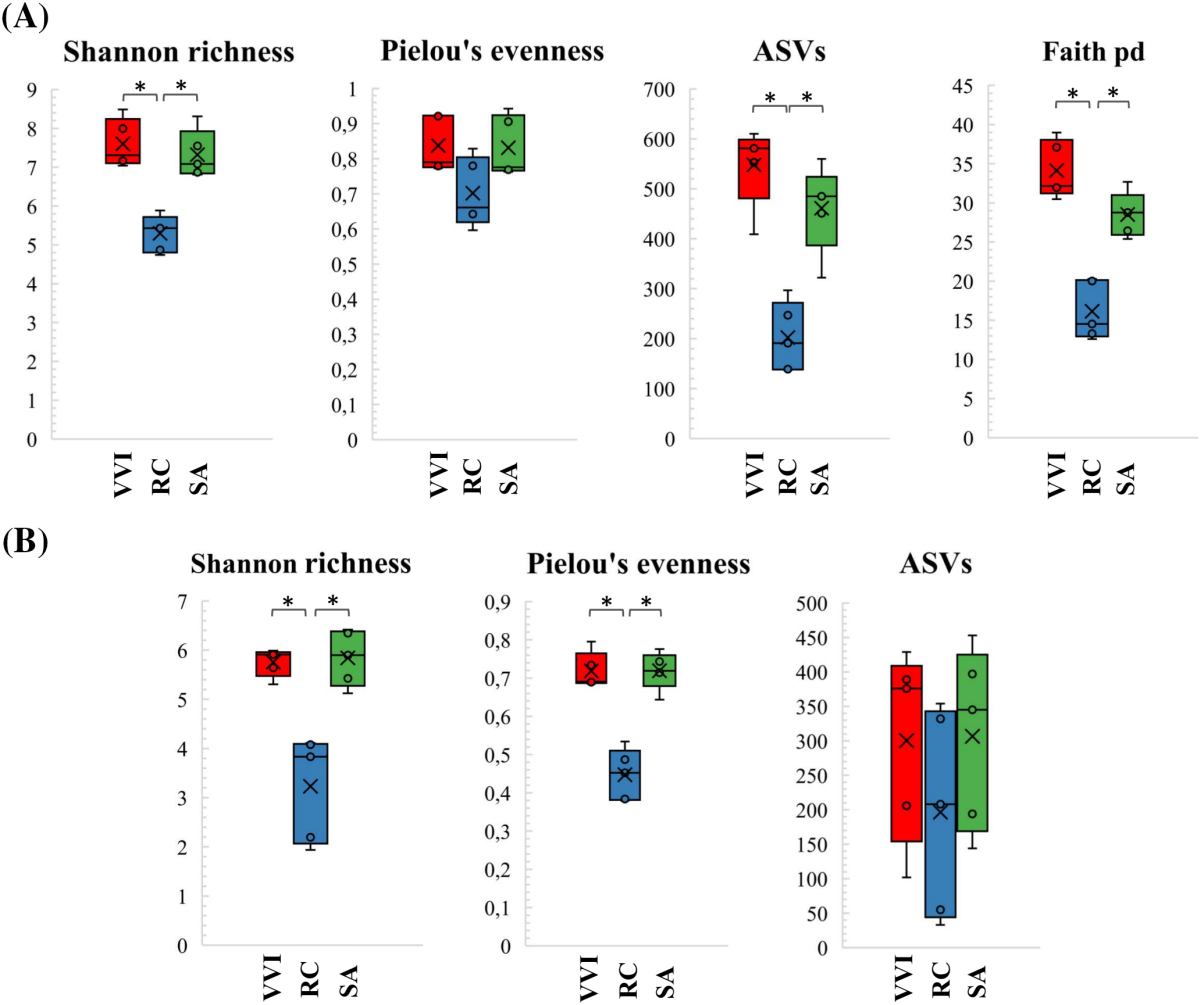
Alpha diversity analysis of lingonberries, rosehips and rowanberries bacterial (A) and fungal (B) microbiota. ASV, Amplicon sequencing variant; RC, rosehip; SA, rowanberry; VVI, lingonberry. The asterisk above the column indicates statistically significant differences (*p* <0.05).

**FIGURE 2:**
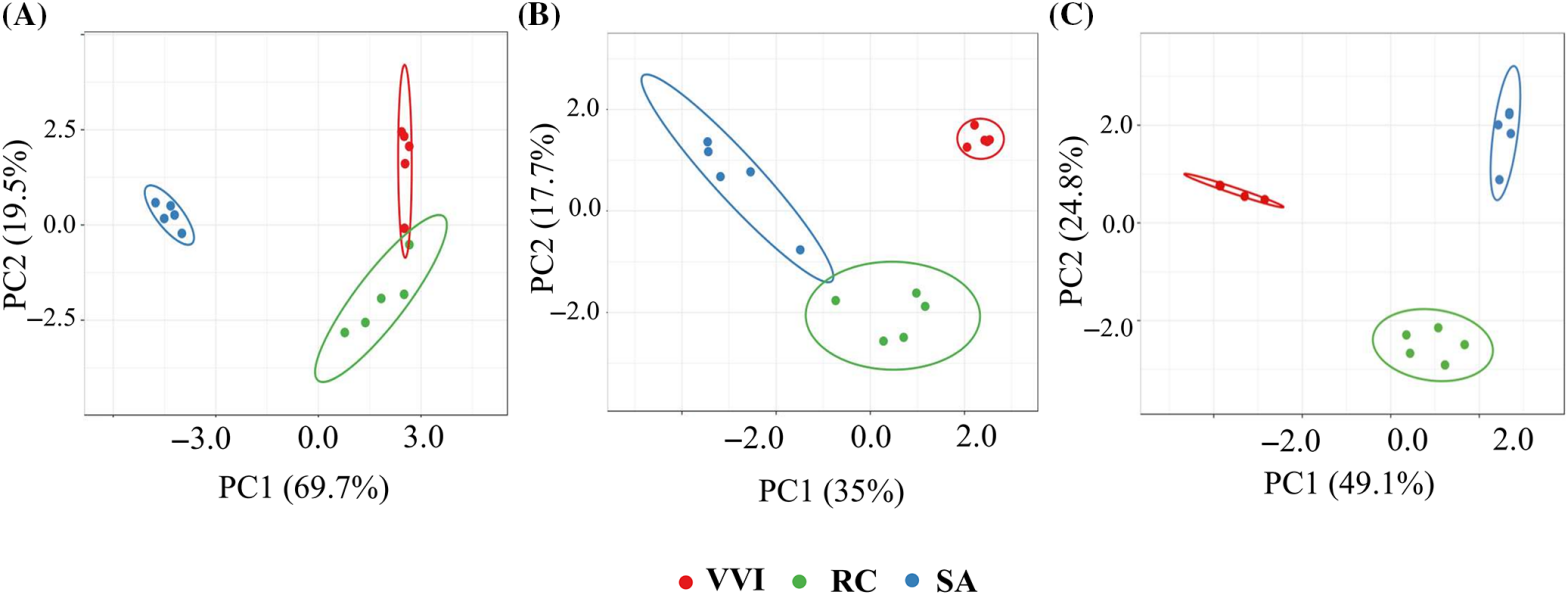
Comparison of bacterial (A, B) and fungal (C) microbiota on lingonberries, rosehips and rowanberries by principal coordinate analysis. Plots were counted using weighted (A) and unweighted (B) UniFrac and Bray‐Curtis (C) distances. RC, rosehip; SA, rowanberry; VVI, lingonberry.

The Shannon's richness index did not reveal statistical differences in microbial diversity among lingonberry (VVI) and rowanberry (SA) samples (Kruskal‐Wallis test for bacteria *p* = 0.347 and fungi *p* = 0.917). However, both differed significantly with the bacterial and fungal diversity observed on rosehip fruits (RC) (*p* <0.05) (Figure 1A,B). The Pielou's evenness index did not show statistical differences within bacterial groups (Figure 1A) but significant differences in fungal diversity on lingonberry versus rosehip (*p* = 0.009) and on rowanberry versus rosehip (*p* = 0.009) were revealed (Figure 1A,B). ASVs and Phylogenetic diversity index (Faith's PD) indicated statistically significant differences between rosehips and lingonberries (*p* = 0.009) and between rosehips and rowanberries (*p* = 0.009) in bacterial samples, while no significant differences were observed in fungal samples (Figure 1A,B). Beta diversity analysis, using weighted and unweighted UniFrac and Bray Curtis metrics, showed significant differences in bacterial and fungal communities across different berries tested, both in terms of species and relative abundances. Principal coordinate analysis (PCoA) based on unweighted and weighted UniFrac distances of bacterial microbiota revealed a separation between lingonberry, rowanberry and rosehip samples (Figure 2A,B). PCoA based on Bray Curtis of the fungal microbiota also showed clear separation of all tested berries (Figure 2C).

Based on the results of the permutational multivariate analysis of variance (pairwise PEMANOVA), there are significant differences in the composition of the bacterial and fungal community between the SA, VVI and RC samples for all beta diversity metrics (UniFrac *p*‐value <0.05) (Table S2). For bacteria, the differences are more pronounced considering feature abundance (weighted UniFrac) when compared to presence/absence (unweighted UniFrac). In VVI versus RC samples, the weighted pseudo‐F value (35.898) is 6.5 times higher than the unweighted pseudo‐F value (5.477), indicating that the difference between these groups is more pronounced when considering feature abundance rather than just presence/absence. Similar results were obtained for VVI versus SA samples (with the weighted pseudo‐F (7.694) being higher than the unweighted pseudo‐F (3.899). RC versus SA weighted pseudo‐F (24.133) is also higher than unweighted pseudo‐F (3.476).

### Bacterial community profiling on lingonberry, rosehip and rowanberry

**FIGURE 3:**
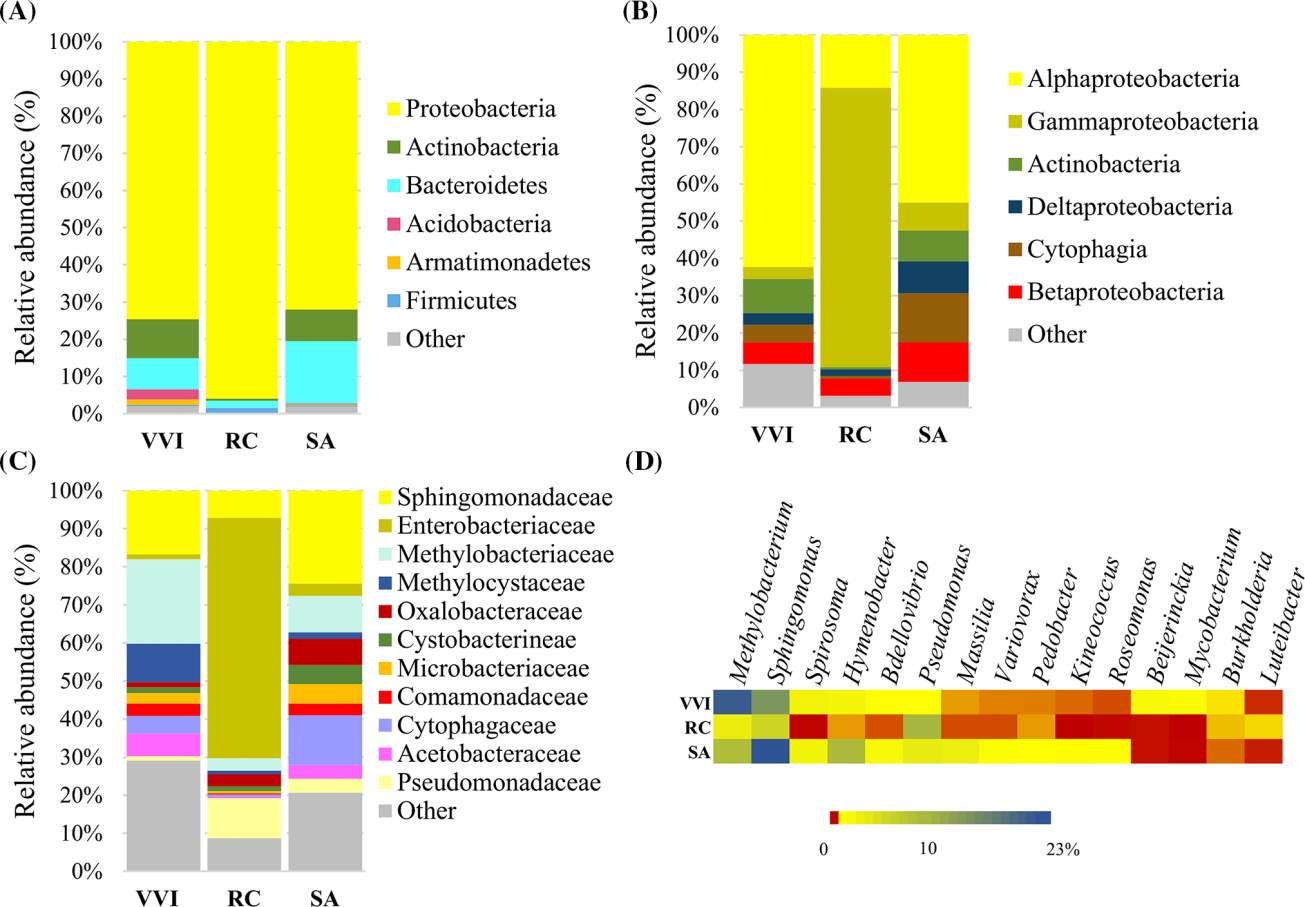
Distribution of bacterial microorganisms on lingonberries, rosehips and rowanberries at phylum (A), class (B) and family (C) levels. (D) Heatmap of the most common genera of bacteria. RC, rosehip; SA, rowanberry; VVI, lingonberry.

Of the 16 bacteria phyla detected on lingonberries, Proteobacteria was the most abundant (74.56%), followed by Actinobacteria (10.46%) and Bacteroidetes (8.39%) (Figure 3A). Among the 32 classes identified, Alphaproteobacteria (62.19%) prevailed (Figure 3B) and was represented mainly by *Methylobacteriaceae* (22.16%), *Sphingomonadaceae* (16.61%) and *Methylocystaceae* (10.12%) at the family level (Figure 3C, Figure S2). The microorganisms of the Actinobacteria, Betaproteobacteria and Cytophagia classes were observed at a lower level (9.16%, 5.91%, 4.63%, respectively) (Figure 3B). At the genus level, 88 bacterial representatives were distributed on lingonberries in the following order by frequency: *Methylobacterium* (22.15%), *Sphingomonas* (14.86%), *Spirosoma* (2.31%), *Hymenobacter* (2.13%) and others (Figure 3D, Table S3).

Analysis of the rosehips‐inhabiting bacterial microbiota revealed that 11 phyla were represented (Table S3). Proteobacteria was the most abundant phylum accounting for more than 95% of the total bacterial population (Figure 3A) and represented by Gammaproteobacteria (74.99%), Alphaproteobacteria (14.15%) and Betaproteobacteria (4.65%) at the class level (Figure 3B). Among 51 families detected on lingonberries, *Enterobacteriaceae* (63.14%) was the most prevalent, followed by *Pseudomonadaceae* (10.53%), *Sphingomonadaceae* (7.10%), *Methylobacteriaceae* (3.26%), *Oxalobacteriaceae* (3.16%) and others (Figure 3C, Figure S2). Rosehip berries‐associated bacterial population consisted of 63 genera, dominated by *Pseudomonas* (10.45%), *Sphingomonas* (6.53%) and *Methylobacterium* (3.24%) (Figure 3D, Table S3).

The rowanberry microbial community was assigned to 17 phyla and dominated by Proteobacteria, comprising 71.91% of the detected taxa. At the same time, Bacteroidetes and Actinobacteria constituted 16.8% and 8.53%, respectively, with smaller contributions from the other phyla (Table S3, Figure 3A). Alphaproteobacteria (44.91%) dominated among 26 identified classes, followed by Cytophagia (13.09%), Betaproteobacteria (10.63%), Deltaproteobacteria (8.64%), Actinobacteria (8.31%), Gammaproteobacteria (7.51%) and others (Figure 3B). Within the 64 identified bacterial families, the notable prevalence was observed in *Sphingomonadaceae* (24.36%), *Cytophagaceae* (13.08%), *Methylobacteriaceae* (9.53%), *Oxalobacteraceae* (6.87%), *Microbacteriaceae* (5.11%) and *Cystobacterineae* (5.01%) (Figure 3C, Figure S2). Among the 74 bacterial genera found on rowanberries, the prominent genera were *Sphingomonas* (23.18%), *Hymenobacter* (9.99%), *Methylobacterium* (9.52%), *Pseudomonas* (3.56%) and *Massillia* (3.09%) (Figure 3D, Table S3).

The bacterial microbiota associated with all tested berries showed differences starting at the higher taxonomic level. On rosehips, Gammaproteobacteria dominated, while on lingonberries and rowanberries, Alphaproteobacteria prevailed. At the family level, more abundant Enterobacteriaceae representatives were found on the rosehips, *Methylobacteriaceae* were detected on lingonberries, and *Sphingomonadaceae*—on rowanberries (Figure 3). The heatmap depicts the distribution of the 15 most abundant bacterial genera (Figure 3D) revealing the differences between all three groups of berries—lingonberry, rosehip and rowanberry. *Methylobacterium* and *Sphingomonas* were the dominant genera in all tested berries, along with *Pseudomonas* on rosehip and *Hymenobacter* on rowanberry. *Spirosoma* and *Bdellovibrio* were common on lingonberries and rowanberries, while *Massilia*, *Variovorax*, *Pedobacter*, *Kineococcus* and *Roseomonas* were found mainly on rowanberries. *Beijerinckia* and *Mycobacterium* were specific to lingonberries, whereas *Luteibacter* was detected on rosehips mainly. *Burkholderia* was found on all tested berries but in different amounts.

### Fungal community profiling on lingonberry, rosehip and rowanberry

**FIGURE 4:**
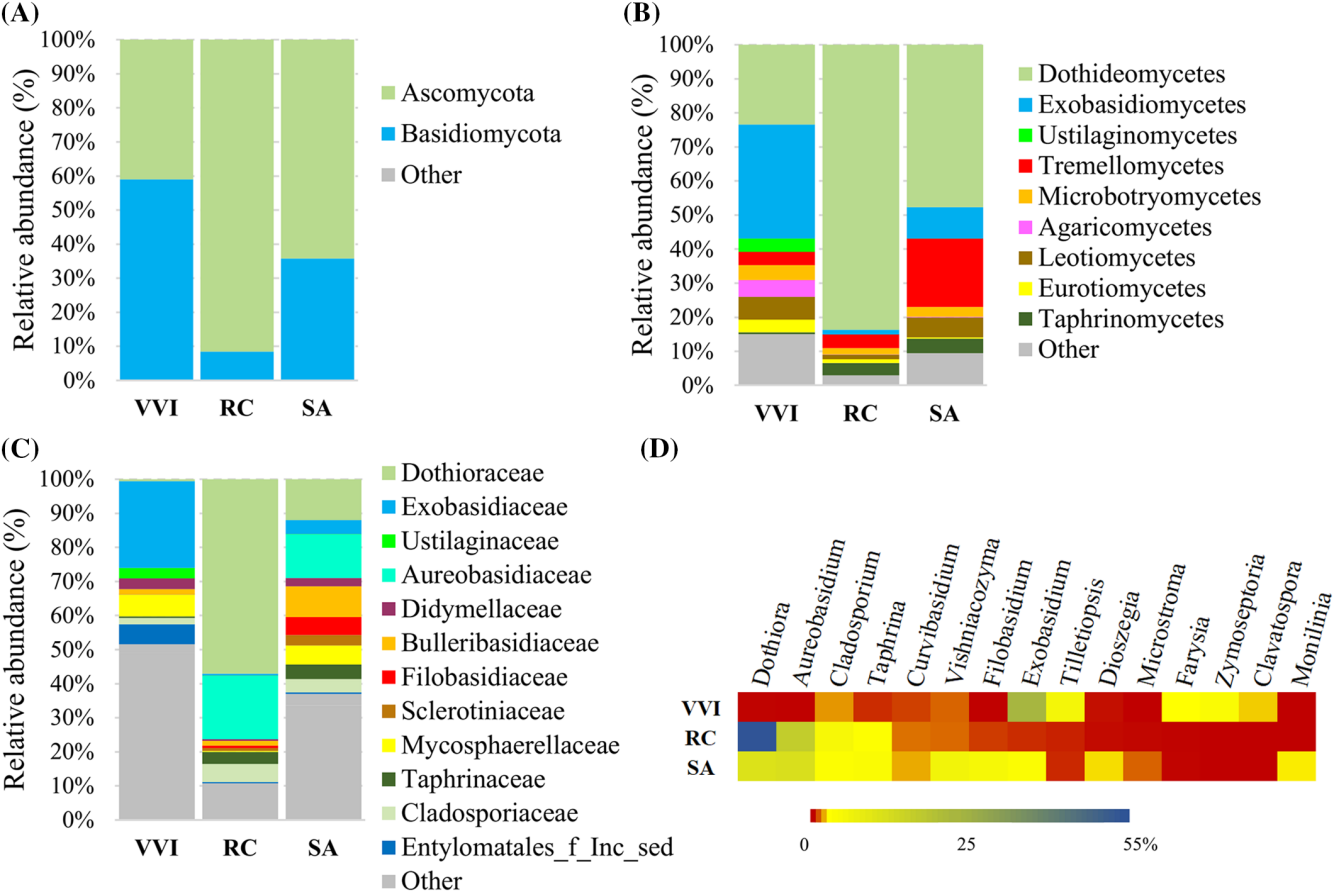
Fungal microbiota on lingonberries, rosehips and rowanberries at phylum (A), class (B) and family (C) levels. (D) Heatmap of the most common genera of fungal microorganisms. RC, rosehip; SA, rowanberry; VVI, lingonberry.

Data on lingonberries‐inhabiting fungal microbiota revealed that Basidiomycota (59.0%) was the dominant phylum, represented mainly by Exobasidiomycetes (33.5%). The second in abundance phylum was Ascomycota (41.0%) (Figure 4A), whose representatives at the class level were assigned to Dothideomycetes (23.4%) and Leotiomycetes (6.7%) (Figure 4B). On lingonberries, differentiated 15 classes were represented by 48 families, dominated by Exobasidiaceae (25.5%) and Mycosphaerellaceae (6.4%) (Figure 4C, Figure S2). Furthermore, the analysis revealed that *Exobasidium* (25.5%), *Tilletiopsis* (5.8), *Zymoseptoria* (4.4%) and *Farysia* (3.1%) are the main fungal genera among all 52 found on lingonberries (Figure 4D, Table S4).

The fungal microorganisms associated with rosehips belonged to 2 phyla, 13 classes, 39 families and 41 genera (Table S4). Ascomycota (91.6%) was the most abundant phylum, while Basidiomycota comprised only 8.4% (Figure 4A). The first phylum was represented by Dothideomycetes (83.6%) and Taphrinomycetes (3.6%), at the class level and the second—by Tremellomycetes (4.0%) and Microbotryomycetes (1.9%) (Figure 4B). About half of the population of whole fungal microorganisms at the family level belonged to Dothioraceae (57.1%) and followed by Aureobasidiaceae (18.7%), Cladosporiaceae (5.3%), Taphrinaceae (3.6%) and others (Figure 4C, Figure S2). These families were represented by fungi of the genera *Dothiora*, *Aureobasidium*, *Cladosporium* and *Taphrina* (Figure 4D, Table S4).

The fungal microbiota of rowanberry surface belonged to 2 phyla: Ascomycota (64.3%) and Basidiomycota (35.7%) (Figure 4A). At the class level, Dothideomycetes (47.7%), Tremellomycetes (20.1%), Exobasidiomycetes (9.2%), Leotiomycetes (5.8%) and Taphrinomycetes (4.3%) were identified as the most abundant (Figure 4B). Of the 56 families detected on rowanberries, eight were comprised with a similar 3% exceeding abundance. Among them, Aureobasidiaceae (12.8%), Dothioraceae (11.9%), Bulleribasidiaceae (9.0%) and Filobasidiaceae (5.3%) were the dominant (Figure 4C, Figure S2), and represented by fungi from the genera *Aureobasidium*, *Dothiora*, *Vishniacozyma* and *Filobasidium* (Figure 4D, Table S4).

The diversity of fungal microorganisms differed among the berries tested. *Dothiora* and *Aureobasidium* were the most abundant fungal genera on rosehip and rowanberry, followed by *Cladosporium* and *Taphrina*. While *Exobasidium* was dominant on lingonberry (Figure 4D). Certain microorganisms, from the *Vishniacozyma*, and *Curvibasidium* genera, were detected in varying amounts on all tested berries but prevailed only on rowanberries. Meanwhile, the representatives of *Dioszegia*, *Microstroma* and *Monilinia* were specific only for the rowanberries.

### Distribution of cultivable yeasts on rowanberries, rosehips and lingonberries

**FIGURE 5:**
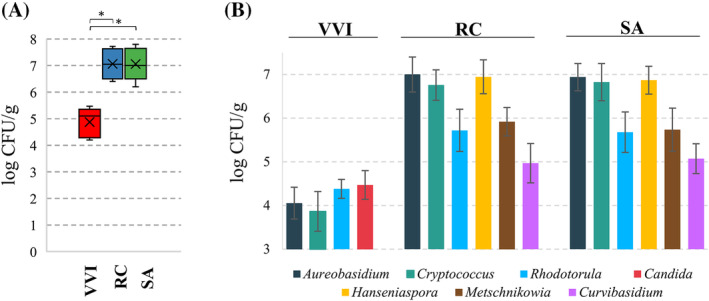
(A) Total count of fungal microorganisms on lingonberry, rosehip and rowanberry. (B) Distribution of fungal microorganisms depending on berries. RC, rosehip; SA, rowanberry; VVI, lingonberry. The asterisk above the column indicates statistically significant differences (*p* <0.05).

The distribution of cultivable yeasts on rowanberries, rosehips and lingonberries was investigated in this study also. It was revealed that the viable yeast populations on rowanberries and rosehips were similar, with 7.058 log CFU/g of berries and 7.062 log CFU/g, respectively (Figure 5A). However, the cultivable yeast population on lingonberries was about 100‐fold lower, with 4.874 log CFU/g, and differed greatly from rowanberries and rosehips.

Morphologically different yeast colonies were identified using molecular methods by sequencing the ITS region. The cultivable yeast profile on rowanberries and rosehips was similar, both featuring the same yeast genera (*Aureobasidium*, *Cryptococcus*, *Curvibasidium*, *Hanseniaspora*, *Metschnikowia* and *Rhodotorula*) with comparable cell counts per gram on both berries (Figure 5B). However, the yeast composition and amounts on lingonberries differed significantly. *Aureobasidium* sp. was about 1000‐fold lower (4.05 ± 0.37 log CFU/g) on lingonberries compared to rowanberries (6.94 ± 0.31 log CFU/g) and rosehips (7.00 ± 0.40 log CFU/g). *Cryptococcus* sp. and *Rhodotorula* sp. were also detected on lingonberries but at approximately 10–100‐fold lower levels than on rosehips and rowanberries. *Candida* sp. (4.47 ± 0.33 log CFU/g) was identified only on lingonberries, a similar level to *Rhodotorula* sp. (4.38 ± 0.22 log CFU/g). Meanwhile, representatives of *Hanseniaspora* and *Metschnikowia* were isolated from rowanberries and rosehips, but not lingonberries. In general, the results of this study suggest that the distribution of cultivable yeasts on rowanberries, rosehips and lingonberries varies significantly, with lingonberries exhibiting a distinct yeast profile compared to the other two berries.

## DISCUSSION

While lingonberries, rowanberries and rosehips are well‐recognized for their health benefits and antimicrobial properties (Bartkiene et al., 2019; Cioch et al., 2017; Ghendov‐Moșanu et al., 2018), detailed insights into their microbial inhabitants remain scarce. Previous studies have identified some endophytes in lingonberries and rosehips and epiphytes on rowanberries (Maksimova et al., 2009; Nguyen et al., 2021; Rovná et al., 2015, 2020), they did not utilize advanced sequencing analysis. Our study aims to fill this gap by characterizing bacterial and fungal communities on the surface of these berries using 16S and ITS2 DNA sequencing. We also employed culture‐dependent methods to provide a more comprehensive understanding of the yeast variety on the surface of berries, offering insights that were previously unattainable with only traditional culture‐dependent methods.

The NGS analysis determined distinct bacterial and fungal communities across the three berries varying significantly. Lingonberry and rowanberry samples exhibited higher microbial diversity and ASV counts than rosehips both in terms of richness and community composition. Beta diversity analysis demonstrated significant differences in both bacterial and fungal communities. The distinct clustering of microbial communities suggests that each berry type supports a unique microbial ecosystem. Substantial differences can be attributed to several factors including environment, nutrient availability, berry surface characteristics and microbial interactions (Vorholt, 2012).

Microbial profiling revealed dominant bacterial phyla such as Proteobacteria, Actinobacteria and Bacteroidetes on lingonberries and rowanberries and Proteobacteria—on rosehips. At the genus level, *Methylobacterium* and *Sphingomonas*, were prevalent across all berry types, while *Pseudomonas* was more abundant on rosehips. On lingonberries, the dominance of Alphaproteobacteria, specifically the *Methylobacteriaceae* and *Sphingomonodaceae* families, suggests a community adapted to utilize methanol and other C1 compounds released by the berry (Asaf et al., 2020; Green & Ardley, 2018). These bacteria are known for their role in plant health and growth promotion by synthesizing antimicrobial metabolites providing lingonberries with a competitive advantage in their natural habitats (Asaf et al., 2020; Sanjenbam et al., 2022). Some species of *Methylobacterium* have been found to have antimicrobial properties and are rarely opportunistic pathogens to humans (Green & Ardley, 2018). *Sphingomonas* spp., similar to *Methylobacterium*, can degrade xenobiotic compounds, herbicides, pesticides and chemical pollutants, therefore have high ecological relevance for use in bioremediation and development of sustainable agriculture (Asaf et al., 2020).

Rowanberries showed a more balanced bacterial population with considerable contributions from Alphaproteobacteria, Betaproteobacteria and Cytophagia. The presence of *Sphingomonas*, *Hymenobacter* and *Methylobacter* suggests a robust microbial community. Bacteria belonging to *Hymenobacter* genera are isolated from diverse habitats, such as air, soil, leaves, various tree barks and other extremely low‐nutrient environments (Cha et al., 2020; Damdintogtokh et al., 2022). The ability of *Hymenobacter* to survive in harsh conditions highlights their potential role in improving the stress tolerance of the rowanberry plant. Additionally, these bacteria can produce carotenoids, which are valuable for their antioxidant properties and possible applications in biotechnology or could be employed in bioremediation processes for agro‐industrial waste management and reduction of environmental pollution (Klassen & Foght, 2008; Maglione et al., 2024).

The bacterial community on rosehips was heavily dominated by the *Enterobacteriaceae* and *Pseudomonodaceae* families, particularly *Pseudomonas* genera. Among bacterial endophytes inhabiting *Rosa canina* L. fruits the high total viable counts of *Enterobacteriaceae* and *Pseudomonas* were also observed (Rovná et al., 2015, 2020). These bacteria are considered an important predictor of food safety, often associated with food spoilage and pathogenicity (Baylis et al., 2011). The high abundance of *Enterobacteriaceae* could indicate a higher susceptibility of rosehips to microbial deterioration and disease, this is consistent with reduced total microbial diversity observed in alpha diversity analysis. For humans, many *Enterobacteriaceae* species are known to cause infections such as urinary tract infections, pneumonia, wounds and blood infections. They are also recognized as a consistent and abundant member of the microbial community in the plant phylosphere, and some members contribute to the suppression of plant disease (Bonaterra et al., 2022; Moreira de Gouveia et al., 2024). *Pseudomona*s have evolved to survive in environments with low nutrient availability and are known to cause various plant diseases, including rots, spots, necrosis of plant parts (Fernández‐Sanz et al., 2022) and human diseases when the normal immune system is compromised (Mulcahy et al., 2014).

Less abundant bacteria such as *Spirosoma*, *Bdellovibrio*, *Massilia*, *Variovorax* and *Beijerinckia* were also identified. Although *Spirosoma* is known to be recovered from various environments (soil, dust, freshwater, etc.) (Li et al., 2018), it has not previously been mentioned as being found on berries. Previously, *Spirosoma* has only been detected by NGS on apple flowers, and passion fruit (Vermote et al., 2022), but in this study, it was observed on lingonberries and rowanberries as well. These bacteria play a vital ecological role by contributing to the decomposition of organic matter and helping with nutrient cycling, particularly carbon and nitrogen, which are essential for plant growth in various habitats (Fries et al., 2013). *Beijerinckia*, specifically noted on low‐growing lingonberries, are ecologically relevant due to their atmospheric nitrogen fixation, ability to promote growth directly by producing growth‐promoting substances and indirectly improving soil nutrient content, and adaptability to harsh environmental conditions (Gamit & Amaresan, 2022; Reiko Sato Miysaka et al., 2003). The natural antibacterial properties of lingonberries and rosehips could be augmented by *Bdellovibrio*, which may act as a potential prebiotic and antibiotic agent against bacterial pathogens and act as a biocontrol agent (Atterbury & Tyson, 2021; Cavallo et al., 2021; Waso et al., 2021). Some species of *Massilia* (more abundant on lingonberries) and *Variovorax* (more abundant on rowanberries) can suppress pathogens for healthy plants (Flores‐Duarte et al., 2022; Ofek et al., 2012). In addition, *Variovorax* can degrade benzene, making members of this genus useful for plants growing in contaminated and poor soil (Flores‐Duarte et al., 2022; Posman et al., 2016). *Mycobacterium* is known for slow growth and resistance through biofilm making in various environments, some species have been shown to promote plant growth under saline conditions (Bouam et al., 2018), and only a few species can cause various human diseases, including tuberculosis, leprosy and lung infections (Tsouh Fokou et al., 2015).

The fungal microbiota associated with lingonberries, rosehips and rowanberries showed quantitative differences at the highest taxonomic level. On lingonberries, the fungal community is dominated by Basidiomycota, particularly Exobasidiaceae. *Exobasidium* fungi with more than 170 species are primarily pathogenic to plants within the *Ericaceae* family, where lingonberries belong (Dong et al., 2019; Ek et al., 2006). This dominance suggests that *Exobasidium* may be a significant factor affecting the health of lingonberry plants, potentially transmitted from damaged leaves even if berries themselves appear visually healthy. Other fungi genera detected on lingonberries include *Tilletiopsis*, *Farysia*, *Zymoseptoria* and *Clavatospora*. Their role in berries is not well defined, but further exploration could lead to novel biocontrol strategies, contributing to the reduction of chemical pesticide use. Some representatives of the *Tilletiopsis* genus are involved in postharvest disorders in apples, posing a threat to humans by causing subcutaneous mycosis and orbital infections (Godfrey et al., 2018). *Zymoseptoria*, identified among the fungal communities, functions as a wheat pathogen, leading to economic losses and there is no information on how it could affect berries or other fruit crops (Torriani et al., 2015).

Basidiomycota and Ascomycota resided on rowanberries, with *Dothiora* and *Aureobasidium* being the most prominent. Many species of *Dothiora* are saprobic, some are pathogens causing leaf spots or other diseases on stressed plant tissues (Senwanna et al., 2024), which could influence the health and growth of rowanberries. Moreover, *Dothiora* sp. is recorded to produce cytotoxic compounds against cancer cell lines (Pérez‐Bonilla et al., 2017). Other notable fungi include *Vishniacozyma* genus. This fungus can form biofilms and protect fruits from *Botrytis cinerea* infections (Gorordo et al., 2022; Nian et al., 2023), as well as humans from indoor exposure to the fungus, causing allergic airway diseases (Rush et al., 2022). Members of the *Curvibasidium* genus are understudied, but due to lipid production with high fractions of oleic and linoleic acids, they have potential applications in biotechnology (Bai et al., 2023). Other fungi contributing to the microbiome of rowanberries include *Filobasidium* (syn. *Cryptococcus*), *Dioszegia*, *Microstroma* and *Monilinia. Filobasidium* is a potentially phytopathogenic, found on apples, pears, cherries, pine trees, junipers and others, capable of producing killer toxins that modulate the microbial community and protect against opportunistic pathogens (Arrigoni et al., 2018; Bao et al., 2022; Oliveira Longa et al., 2022; Stanevičienė et al., 2021; Vepštaitė‐Monstavičė et al., 2018). *Dioszegia* is a useful genus that can directly interact with phyllosphere bacteria, affecting their diversity and composition (Agler et al., 2016). *Microstroma* causes spots and blotches (Frank et al., 2010; Lutz et al., 2018), while *Monilinia* triggers rots of apples, plums, sweet cherries and others (De Miccolis Angelini et al., 2022; Deltedesco et al., 2023; Rosati et al., 2012). The growth of phytopathogen *Monilinia* can be suppressed by *Pseudomonas* sp. (Kolytaitė et al., 2022).

Rosehips were mainly inhabited by Ascomycota, with *Dothiora* and *Aureobasidium* being the two leading genera. *Aureobasidium* genus is known for its wide range of extracellular enzymes, and metabolic capabilities, including the degradation of various organic substances, which could be beneficial for rosehips in low‐nutrient environments (Wang et al., 2022). Other detected fungi are *Cladosporium* and *Taphrina*, which often include plant pathogens causing tumours and lesions, and hyperparasite other fungi (Sandoval‐Denis et al., 2016; Tsai et al., 2014). Certain species are relevant as potential biocontrol agents, especially *Cladosporium* which can produce secondary metabolites active against *Bacillus subtilis* and *Escherichia coli* (AlMatar & Makky, 2016). Spores of some species of *Cladosporium* can cause allergies, posing a risk to human health (Sandoval‐Denis et al., 2016). Representatives of *Cladosporium* genera were also detected among endophytic fungal microorganisms inhabiting rosehips and other wild berries, such as blueberries (Rovná et al., 2020; Sun et al., 2023).

For a comprehensive assessment of wild berries‐inhabiting microbial communities, cultivable yeasts were analysed. Comparable cultivable yeast profiles were obtained for rowanberries and rosehips. Detected representatives of *Aureobasidium*, *Cryptoccocus*, *Rhodotorula*, *Hanseniaspora*, *Metschnikowia* and *Curvibasidium* genera were found on both berries. These data correspond to previous rowanberry epiphytic microbiota studies (Maksimova et al., 2009). Lingonberries differed in the quantity and composition of cultivable yeasts, thus illustrating unique microbial environments. *Aureobasidium* and *Cryptococcus* were about 1000‐fold lower on lingonberries than on the other two berries. *Aureobasidium* was also found among endophytic *Vaccinium vitis‐idaea* L. colonizing yeast strains (Nguyen et al., 2021). *Rhodotorula* spp. yeasts were present at more than 10‐fold lower levels on lingonberries. In our study, *Candida* spp. was uniquely found on lingonberries at levels similar to other detected yeasts. However, others detected *Candida* spp. and *Rhodotorula* spp. among endophytic yeasts inhabiting rosehips (Rovná et al., 2020). *Rhodotorula*, *Hanseniaspora*, *Metschnikowia* and *Curvibasidium* were found on rosehips and rowanberries, but not on lingonberries. These data correspond to metagenomics results which show no or only a few reads of these genera. The dominance of *Aureobasidium* and *Cryptococcus* on lingonberries, but the absence of *Hanseniaspora* and *Metschnikowia*, which are typically more prevalent during maturation stages (Barata et al., 2012; Lukša et al., 2020), suggests that they were likely not fully ripe. While culture‐based methods provide valuable insights into the viable yeast populations on berries, they are inherently biased toward microorganisms that can grow under the specific culture conditions applied. These methods often fail to capture the full diversity of yeast species, especially those that are slow‐growing or require specific nutrients and conditions not provided in our media. As a result, certain yeast species, particularly those that are not readily culturable, may have been underrepresented or missed entirely in our analysis. Understanding these microbial dynamics can inform harvesting practices and post‐harvest treatment to improve berry quality and shelf life. Obtained data suggest lingonberries may have more robust antimicrobial properties or less favourable conditions for yeast growth, possibly due to higher levels of phytochemicals or a more resilient surface structure (Kowalska, 2021).

Our research has broader implications in the context of food safety. Various organizations (e.g., the World Health Organization, the Food and Agriculture Organization of the United Nations and others) and nutrition experts encourage the consumption of wild berries (Newman, 2021). Given the preference to consume unprocessed berries, monitoring their microbiological status is of great importance (Oliveira et al., 2019). Great effort is required to ensure quality and food safety control; thus, the implementation of modern techniques offers a solution to mitigate the risks. The microbial communities identified on lingonberries, rowanberries and rosehips present important food safety considerations, particularly regarding the presence of potential pathogens. Rosehips exhibited a high abundance of *Enterobacteriaceae* and *Pseudomonas*, both of which are known as opportunistic pathogens and indicators of food spoilage and human pathogenicity (Baylis et al., 2011; Fernández‐Sanz et al., 2022). In addition, representatives of the *Enterobacteriaceae* family and *Pseudomonas* genera are known to be involved in the development and spreading of multidrug resistance (Mancuso et al., 2021). Fungi are considered the dominant group of microorganisms causing deterioration of food products and posing serious threats to human health (Sun et al., 2023). *Cladosporium*, a fungi genus found predominantly on rosehips, is commonly associated with food spoilage, particularly in refrigerated environments. While *Cladosporium* does not produce harmful mycotoxins, its presence can lead to visible mould growth, off‐flavours and potential allergic reactions (Pouris et al., 2024). Proper refrigeration and moisture control are therefore essential to prevent contamination and extend the shelf life of products. The presence of these microorganisms suggests rosehips may have a higher susceptibility to microbial deterioration and could pose a greater food safety risk if not properly handled or processed. In contrast, lingonberries and rowanberries, with their dominant populations of *Methylobacterium* and *Sphingomonas*, may offer natural antimicrobial protection due to their ability to produce bioactive compounds, thereby extending shelf life and reducing the risk of spoilage (Asaf et al., 2020; Sanjenbam et al., 2022). However, the identified *Candida* yeasts on lingonberries may pose health risks for immunocompromised individuals when consuming unprocessed berries (Pouris et al., 2024). These findings suggest that while wild berries offer health benefits, proper hygiene and post‐harvest handling are important to prevent microbial contamination and prolong shelf life. Adopting processing strategies such as washing, drying and possibly mild heat treatments could help minimize these risks, ensuring safer consumption of these nutritionally rich berries.

## CONCLUSION

Overall, this study constituted the first NGS‐based analysis of microbial assemblages on rowanberries, rosehips and lingonberries and identified distinct wild berry‐defined bacterial and fungal communities. The lingonberry was dominated by *Methylobacteriaceae* and rowanberries—by *Sphingomonadaceae* representatives, which were known to have high ecological relevance. *Enterobacteriaceae* was found in the highest abundance on rosehips, highlighting food safety concerns while consuming unprocessed berries. Significant differences in fungal community composition across tested berries were observed. Lingonberries prevailed by Exobasidiaceae and rosehips—by Dothioraceae representatives, often associated with plant pathology processes. Many fungal microorganisms, linked to food and human safety, have been detected on rowanberries. The lowest and significantly different culturable yeast population compared to other tested berries was distinguished on lingonberries. The above findings validate our hypothesis that wild berries support unique microbial ecosystems, which may significantly contribute to their health‐promoting properties. The identification of both beneficial and potentially pathogenic microorganisms highlights the importance of microbial diversity in determining the safety and nutritional value of the berries. Omics‐based and culture‐dependent approaches allowed us to attain a comprehensive understanding of the structure of microbial communities associated with tested health‐relevant wild berries, deepen knowledge of their ecological potential and contribute to uncovering their attractiveness for plant and human health. Further studies are needed to investigate the precise functional roles of wild berries‐associated microorganisms and their interactions with the host plants.

## AUTHOR CONTRIBUTIONS

**Iglė Vepštaitė‐Monstavičė:** Writing – original draft; methodology; investigation; visualization; formal analysis. **Juliana Lukša:** Writing – original draft; visualization; software; formal analysis. **Živilė Strazdaitė‐Žielienė:** Methodology; investigation. **Saulius Serva:** Conceptualization; resources; writing – review and editing; funding acquisition. **Elena Servienė:** Conceptualization; resources; methodology; writing – review and editing; data curation; formal analysis.

## CONFLICT OF INTEREST STATEMENT

The authors declare no conflict of interest.

## Supporting information


**Figure S1.** Berries sampling sites in Vilnius district (Lithuania) in 2022. GPS coordinates: VVI1 54°48′0.2″ N, 25°18′30.3″ E; VVI2 54°47′40.4″ N, 25°18′41.7″ E; VVI3 54°47′35.0″ N, 25°18′57.3″ E; VVI4 54°46′19.8″ N, 25°18′47.3″ E; VVI5 54°46′17.4″ N, 25°18′55.6″ E; RC1 54°44′24.3″ N, 25°23′29.8″ E; RC2 54°44′10.8″ N, 25°23′25.7″ E; RC3 54°44′11.3″ N, 25°23′44.9″ E; RC4 54°44′16.9″ N, 25°24′20.1″ E; RC5 54°44′6.9″ N, 25°24′42.1″ E; SA1 54°44′9.9″ N, 25°25′20.6″ E; SA2 54°44′11.2″ N, 25°25′42.5″ E; SA3 54°44′6.2″ N, 25°26′6.9″ E; SA4 54°48′14.6″ N, 25°18′53.7″ E; SA5 54°48′8.5″ N, 25°19′42.2″ E. RC (blue), rosehip; SA (green), rowanberry; VVI (red), lingonberry.

**Figure S2.** Relative abundance of bacterial (A) and fungal (B) microorganisms on all samples of lingonberries, rosehips and rowanberries at family, class and phylum levels. RC, rosehip; SA, rowanberry; VVI, lingonberry.

**Table S1.** Total sequences obtained for eukaryotic (ITS2) and prokaryotic (V3‐V4) microbial community for rowanberry, lingonberry, and rosehip samples. Unused for the eukaryotic community.

**Table S2.** Beta diversity analysis based on weighted and unweighted UniFrac distance metrics for bacteria and Bray‐Curtis for fungi. RC, rosehip; SA, rowanberry; VVI, lingonberry.

**Table S3.** Bacterial taxonomy abundance count of lingonberries (VVI), rosehips (RC) and rowanberries (SA) samples from phylum to species level.

**Table S4.** Yeast taxonomy abundance count of lingonberries (VVI), rosehips (RC) and rowanberries (SA) samples from phylum to species level.

## Data Availability

All data generated or analyzed during this study are included in this article and its supplementary files. The raw sequence data are available in the NCBI Short Read Archive (accession PRJNA1128889).
