# Supplementary figures and images for: Distinct microbial communities associated with health‐relevant wild berries
Source: Environ Microbiol Rep. 2024 Nov 14;16(6):e70048. doi: 10.1111/1758-2229.70048 (PMC11561701; doi:10.1111/1758-2229.70048)

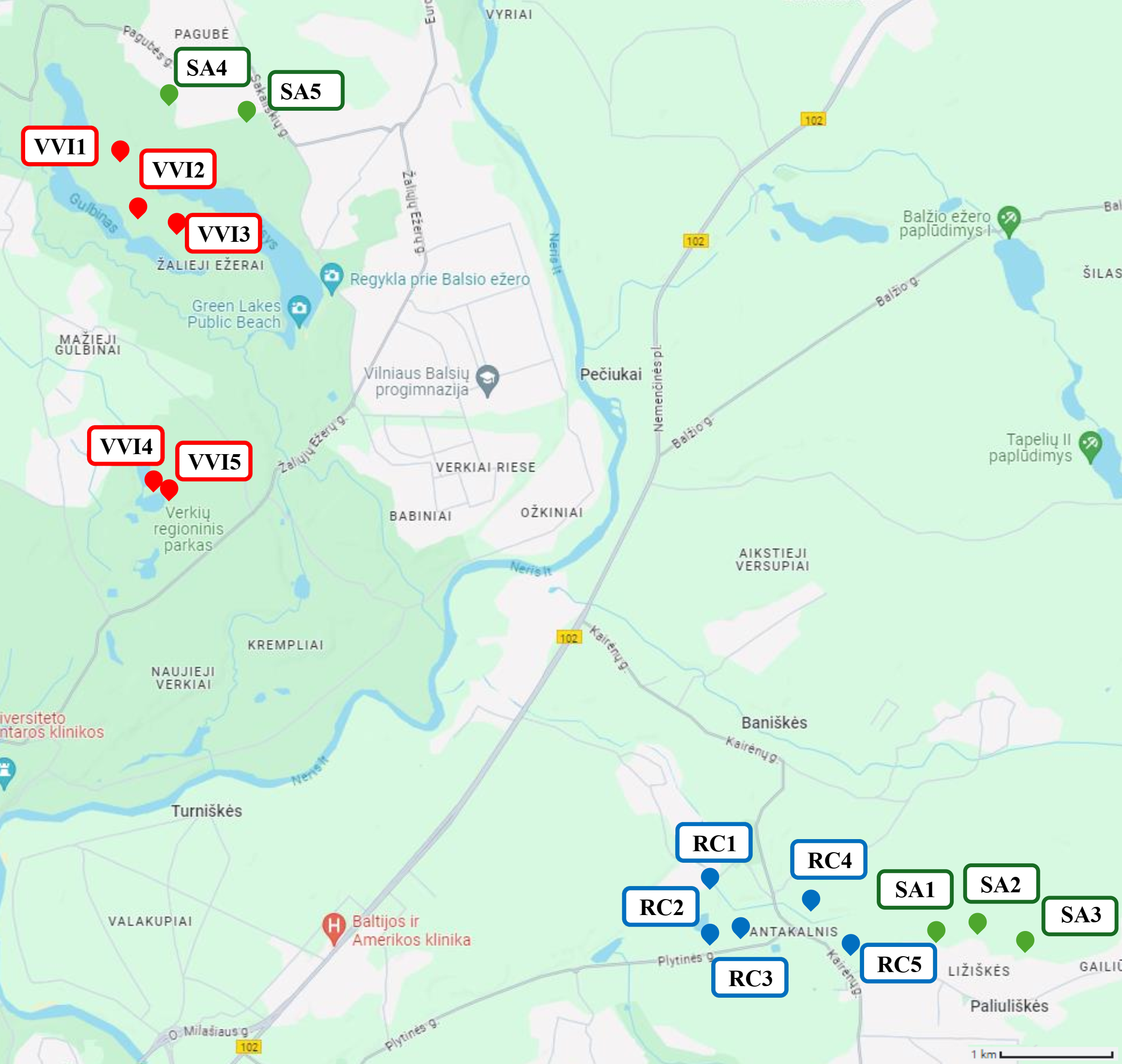

Supplement: Supplementary file 1 — Figure S1. Berries sampling sites in Vilnius district (Lithuania) in 2022. GPS coordinates: VVI1 54°48′0.2″ N, 25°18′30.3″ E; VVI2 54°47′40.4″ N, 25°18′41.7″ E; VVI3 54°47′35.0″ N, 25°18′57.3″ E; VVI4 54°46′19.8″ N, 25°18′47.3″ E; VVI5 54°46′17.4″ N, 25°18′55.6″ E; RC1 54°44′24.3″ N, 25°23′29.8″ E; RC2 54°44′10.8″ N, 25°23′25.7″ E; RC3 54°44′11.3″ N, 25°23′44.9″ E; RC4 54°44′16.9″ N, 25°24′20.1″ E; RC5 54°44′6.9″ N, 25°24′42.1″ E; SA1 54°44′9.9″ N, 25°25′20.6″ E; SA2 54°44′11.2″ N, 25°25′42.5″ E; SA3 54°44′6.2″ N, 25°26′6.9″ E; SA4 54°48′14.6″ N, 25°18′53.7″ E; SA5 54°48′8.5″ N, 25°19′42.2″ E. RC (blue), rosehip; SA (green), rowanberry; VVI (red), lingonberry. [file EMI4-16-e70048-s001.tif]

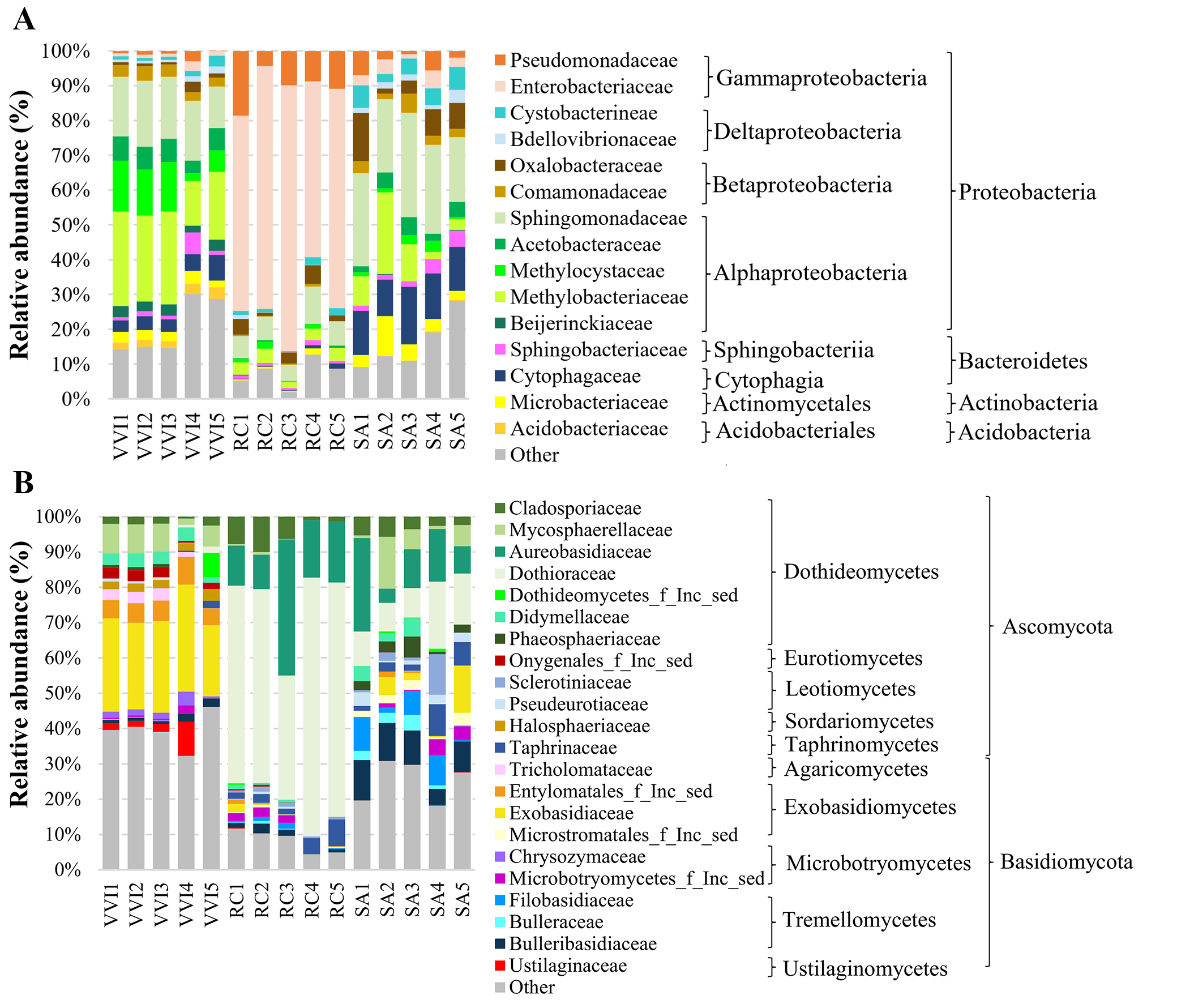

Supplement: Supplementary file 2 — Figure S2. Relative abundance of bacterial (A) and fungal (B) microorganisms on all samples of lingonberries, rosehips and rowanberries at family, class and phylum levels. RC, rosehip; SA, rowanberry; VVI, lingonberry. [file EMI4-16-e70048-s004.tif]
