# Supplementary material for: Distinct microbial communities associated with health‐relevant wild berries
Source: Environ Microbiol Rep. 2024 Nov 14;16(6):e70048. doi: 10.1111/1758-2229.70048 (PMC11561701; doi:10.1111/1758-2229.70048)
Supplement: Supplementary file 3 — Table S1. Total sequences obtained for eukaryotic (ITS2) and prokaryotic (V3‐V4) microbial community for rowanberry, lingonberry, and rosehip samples. Unused for the eukaryotic community. [file EMI4-16-e70048-s003.docx]

| Samples | Target region | High quality reads | ASVs | Pielou evenness | Faith pd | Shannon diversity |
| --- | --- | --- | --- | --- | --- | --- |
| SA1 | ITS2 | 137702 | 345 | 0.64 | - | 5.43 |
|  | V3-V4 | 53813 | 485 | 0.76 | 26.44 | 6.81 |
| SA2 | ITS2 | 138359 | 397 | 0.74 | - | 6.42 |
|  | V3-V4 | 55396 | 560 | 0.78 | 32.69 | 7.08 |
| SA3 | ITS2 | 122406 | 453 | 0.72 | - | 6.34 |
|  | V3-V4 | 51452 | 488 | 0.77 | 29.31 | 6.87 |
| SA4 | ITS2 | 23589 | 144 | 0.71 | - | 5.12 |
|  | V3-V4 | 10720 | 322 | 0.91 | 25.38 | 7.55 |
| SA5 | ITS2 | 25660 | 194 | 0.78 | - | 5.90 |
|  | V3-V4 | 15614 | 451 | 0.94 | 28.76 | 8.31 |
| VVI1 | ITS2 | 98857 | 389 | 0.67 | - | 5.93 |
|  | V3-V4 | 38429 | 553 | 0.77 | 32.17 | 7.04 |
| VVI2 | ITS2 | 146302 | 429 | 0.68 | - | 5.99 |
|  | V3-V4 | 44340 | 610 | 0.79 | 31.94 | 7.31 |
| VVI3 | ITS2 | 104480 | 376 | 0.69 | - | 5.91 |
|  | V3-V4 | 44509 | 587 | 0.78 | 37.10 | 7.17 |
| VVI4 | ITS2 | 38957 | 206 | 0.73 | - | 5.64 |
|  | V3-V4 | 23609 | 581 | 0.92 | 38.98 | 8.49 |
| VVI5 | ITS2 | 14032 | 102 | 0.80 | - | 5.31 |
|  | V3-V4 | 18070 | 409 | 0.92 | 30.46 | 8.00 |
| RC1 | ITS2 | 131959 | 332 | 0.49 | - | 4.08 |
|  | V3-V4 | 41424 | 297 | 0.66 | 20.28 | 5.43 |
| RC2 | ITS2 | 188274 | 354 | 0.45 | - | 3.83 |
|  | V3-V4 | 39478 | 247 | 0.60 | 20.01 | 4.74 |
| RC3 | ITS2 | 150376 | 208 | 0.53 | - | 4.11 |
|  | V3-V4 | 39384 | 191 | 0.64 | 12.60 | 4.87 |
| RC4 | ITS2 | 21234 | 33 | 0.38 | - | 1.94 |
|  | V3-V4 | 6432 | 137 | 0.83 | 14.54 | 5.88 |
| RC5 | ITS2 | 28506 | 55 | 0.38 | - | 2.19 |
|  | V3-V4 | 7158 | 139 | 0.78 | 13.29 | 5.55 |
|  |  |  |  |  |  |  |

**Table 1.** Total sequences obtained for eukaryotic (ITS2) and prokaryotic (V3-V4) microbial community for rowanberry, lingonberry and rosehip samples

- unused for eukaryotic community
