# Supplementary material for: Distinct microbial communities associated with health‐relevant wild berries
Source: Environ Microbiol Rep. 2024 Nov 14;16(6):e70048. doi: 10.1111/1758-2229.70048 (PMC11561701; doi:10.1111/1758-2229.70048)
Supplement: Supplementary file 4 — Table S2. Beta diversity analysis based on weighted and unweighted UniFrac distance metrics for bacteria and Bray‐Curtis for fungi. RC, rosehip; SA, rowanberry; VVI, lingonberry. [file EMI4-16-e70048-s006.docx]

**Table S2.** Beta diversity analysis based on weighted and unweighted UniFrac distance metrics for bacteria (A) and Bray-Curtis for fungi (B). VVI – lingonberry, RC – rosehip, SA – rowanberry.

|  | Weighted Pseudo-*F* | UniFrac  *p*-value | Unweighted Pseudo-*F* | UniFrac  *p*-value |
| --- | --- | --- | --- | --- |
| VVI vs. RC | 35.898 | 0.015 | 5.477 | 0.007 |
| VVI vs. SA | 7.694 | 0.013 | 3.899 | 0.007 |
| RC vs. SA | 24.133 | 0.01 | 3.476 | 0.007 |

**A.**

**B.**

|  | Bray-Curtis | UniFrac  *p*-value |
| --- | --- | --- |
| VVI vs. RC | 17.884 | 0.011 |
| VVI vs. SA | 8.791 | 0.011 |
| RC vs. SA | 7.473 | 0.009 |
